# An assessment of anxiety and depression among HIV-positive pregnant women in a tertiary hospital located in southeast Nigeria: a cross-sectional comparative analysis

**DOI:** 10.3389/fpsyt.2026.1580635

**Published:** 2026-03-06

**Authors:** Chidebe Christian Anikwe, Osita Samuel Umeononihu, Ifeyinwa Helen Anikwe, Cyril Chijioke Ikeoha, Arinze Chidiebele Ikeotuonye, Victor Nwabunwanne Oguaka, Chukwuemeka Jude Ofojebe, Chukwunonso Isaiah Enechukwu, Chidubem Philip Osuagwu, Nwabunike Ekene Okeke, Richard Lawrence Ewah, Mbanefo Paul Okeke

**Affiliations:** 1 Department of Obstetrics and Gynecology, Nnamdi Azikiwe University Teaching Hospital, Nnewi, Anambra, Nigeria; 2 Department of PCR, HIV CARE, Nnamdi Azikiwe University Teaching Hospital, Nnewi, Anambra, Nigeria; 3 Department of Obstetrics and Gynaecology, Alex Ekwueme Federal University Teaching Hospital Abakaliki, Abakaliki, Ebonyi, Nigeria; 4 Department of Anesthesiology, Alex Ekwueme Federal University Teaching Hospital Abakaliki, Abakaliki, Ebonyi, Nigeria

**Keywords:** anxiety, depression, HIV, mental health, pregnancy

## Abstract

**Background:**

The periods of pregnancy are critical for the mental well-being of women. HIV positive pregnant women are especially vulnerable to experiencing depression and anxiety.

**Aim:**

To determine the prevalence and determinant of depression and anxiety among HIV-positive and HIV negative pregnant women in Nnamdi Azikiwe University Teaching Hospital (NAUTH) Nnewi, Nigeria.

**Materials and methods:**

Between January 1 and May 30, 2024, 342 HIV-positive pregnant individuals and an equivalent number of HIV-negative prenatal attendees participated in a cross-sectional survey conducted at NAUTH in Nnewi. A structured questionnaire, the Generalized Anxiety Disorder Assessment, and the Patient Health Questionnaire were used to interview the subjects. IBM Statistical Package for Social Science version 26 was used to analyze the data, and a significance level of less than 0.05 was chosen.

**Results:**

The research involved 684 participants, all of whom screened positive for anxiety and depression. The average scores for anxiety in HIV-positive and HIV-negative women were 16.8 ± 3.8 compared to 8.7 ± 2.3; P <0.001, while for depression the scores were 11.1 ± 4.3 versus 3.1 ± 3.3; P < 0.001, respectively. A significant presence of major depressive and anxiety disorders was discovered among HIV-positive women, with moderate and severe depression affecting 47.7% and 21.9%, respectively, while moderate and severe anxiety were observed in 21.3% and 73.6% of the women. The majority of women in the control group exhibited mild mental health disorders. For HIV-positive women, the significant factors influencing depression included being 30 years old or younger, having a gestational age of 30 weeks or less, possessing a lower educational level, being employed, and being married; for anxiety, key factors were being para 0-3, experiencing psychological IPV, and being married. Among HIV-negative women, significant determinants of depression included being 30 years old or younger, having a low educational level, and being married.

**Conclusion:**

The prevalence of mental health disorders in the study group is extremely high. The rate is unacceptably elevated among pregnant women who are HIV-positive. This emphasizes the need to integrate mental health services into standard maternal healthcare for all women, especially those living with HIV.

## Introduction

Anxiety and depression are the two most common mental disorder that can occur in adult (1; World Health Organization. Anxiety disorders, 2023). Anxiety is a feeling of fear, panic, dread and uneasiness (World Health Organization. Anxiety disorders, 2023). Depression on the other hand is a mood disorder characterized by depressed mood, loss of pleasure and interest in activities for long periods (2). Globally, depression occurs in more than 280 million adults while anxiety disorders was estimated to affect more than 300 million people with both mental disorder more common in women and could lead to suicide ideation (1; World Health Organization. Anxiety disorders, 2023).

The burden of mental disorder during pregnancy is very high and it is a public health problem of global concern. Mental disorders affect roughly 10% of pregnant women and 13% of women who have recently given birth worldwide. This percentage is much higher in developing nations, estimated at 15.6% during pregnancy and 19.8% following childbirth (3). The occurrence of mental disorder varies among pregnant women living or not living with HIV infection (4). This is anticipated since HIV infection comes with numerous psychological challenges (5). Various studies in Africa gave credence to increase number of pregnant HIV women suffering from either depression or anxiety (6–9). In Sub-Saharan Africa, HIV-positive pregnant women (HPPW) had a pooled prevalence of prenatal depression of 30.6% (95% CI, 19.8%-41.3%) (10). In a comparable study, Boakye et al. found that HIV-positive women have a higher burden of worry, ranging from 28.9 to 61% (7). This, invariably, increases the load in pregnancy.

Prenatal psychological adversity can be caused by a variety of factors. Some of the identifiable factors increasing its (mental disorder) occurrence among HIV positive women include: being unmarried, presence of intimate partner violence, stigmatization and history of mental disorder predating pregnancy (10). Other associated factors are social class of the couple, history of bad obstetric outcome, negative life event, lack of formal education, use of alcohol and unplanned pregnancy (9–14). Mental disorder coexisting with pregnancy is associated with deleterious effect on maternal and neonatal outcomes. This is likely to be more among pregnancy complicated with HIV infection (7). HPPW with mental disorder is more at risk of non-compliance with anti-retroviral therapy (10) which if not checked will increase the rate of neonatal vertical transmission. The latter occurrence negates the fight to reduce global increase rate of HIV infection (15). Mental disorder during pregnancy increases the risk of perinatal complications such as preterm labor and delivery (16, 17). Preterm labor and delivery will result in low birth weight and its associated complications (18). Comorbid anxiety and depression in a mother can lead to poor infant cognitive development and mental health problems in later childhood (16). The risk of postpartum psychosis is high (19), limiting mothers’ ability to care for herself and their newborns. The family will suffer, and at the extreme could lead to infanticide and suicide (20).

In Nigeria, the burden of HIV infection is very high (21–23). It was estimated to have affected 1.9 million people with higher percentage in women of reproductive age (22). This increases the risk of mental disorder in pregnancy with its complications (3). In Nigeria, the majority of works on mental disorders in pregnancy analyze solely the prevalence of depression or anxiety states and their associations (13) To the best of our knowledge, there is little research comparing mental disorders in HPPW to a control group without the disease. We propose that HPPW have an increased risk of developing a mental condition. The study’s goal is to investigate the prevalence of depression and anxiety, as well as their drivers, among HIV and non-HIV antennal attendees at Nnamdi Azikiwe University Teaching Hospital in Nnewi, Nigeria. Identifying these women with mental disorder will aid in proper treatment of these women in order to avoid mental disorder sequelae and will help to address a knowledge vacuum in the research domain.

## Materials and methods

### Study design

This study is a cross‐sectional comparative analytical study that assessed the prevalence and determinants of anxiety and depression among HIV-positive pregnant women and a control group of HIV-negative pregnant women attending antenatal care in a tertiary hospital in Nnewi.

### Study setting

The study was carried out in the Department of Obstetrics and Gynaecology of NAUTH, Nnewi between January 1 and May 30, 2024. NAUTH is one of the specialist teaching hospital in the state, receiving referrals from private and mission hospitals from within the state and from neighboring states. The antenatal clinic is held daily on Mondays through Fridays, as were the booking and postnatal clinics. The clinics are ran by consultant obstetricians with their teams of resident doctors and are assisted by nurses. Health talks were given that covered various topical issues including nutrition, diet, personal and environmental hygiene, danger signs during pregnancy, the experience of labor, care of the newborn, exclusive breastfeeding, and immunization. Other health issues, such as hypertension, diabetes mellitus, malaria, anemia, HIV/AIDS, and family planning were also discussed. Baseline investigations requested at booking include: pack cell volume, Hepatitis B surface antigen, Venereal Disease Research Laboratory, Hepatitis C virus, HIV screening after counselling with option to opt out, Blood group, genotype, and urinalysis. Women who screened positive to HIV are further asked to run the following investigations, which are free: serum electrolyte, urea and creatinine, liver function test, viral load, and CD4^+^ count. Folic acid, ferrous sulfate, intermittent prophylactic treatment for malaria using a combination of suiphadoxine-480mg/Pyrimethamine – 25mg (not given to HPPW), and multivitamin supplementation are prescribed. Antiretroviral therapy and Septrin 960mg were also given to HPPW and breast feeding options discussed.

### Study population

The study population comprised HIV positive and HIV negative pregnant women that attended their routine visit to the antenatal clinic (ANC). Consecutive HIV - positive and HIV- negative women who presented for their ANC were recruited. This was continued until the sample size was obtained. Resident doctors (five senior registrars) in the Department of Obstetrics and Gynaecology assisted in data collection. They were informed about the study approach and the study instruments were thoroughly discussed. The chief researcher calculated the total score for anxiety or depression. The study population were matched for age (X years ±2 years) and gestational age (Xweeks ± 2 weeks).

### Study instrument and data source

The study population was interviewed in a dedicated office with a pretested interviewer-administered structured questionnaire, in addition with Patient Health Questionnaire (PHQ-9), Generalized Anxiety Disorder Assessment (GAD-7), and Composite Abuse Scale. The PHQ-9 is the depression module, which scores each of the 9 DSM-IV criteria as “0” (not at all) to “3” (nearly every day). PHQ-9 is good in making diagnoses of depressive disorders, and it is a reliable and valid measure of depression severity (24). Total scores of 5, 10, ≥15 represent cut points for mild, moderate, and severe depression, respectively. GAD-7 is a seven-item instrument that is used to measure or assess the severity of generalized anxiety disorder (25). Each questionnaire took 5–10 minutes to complete. Scores of 5, 10, and ≥15 were taken as the cut-off points for mild, moderate and severe anxiety, respectively.

Both the PHQ-9 and GAD-7 were validated among 50 pregnant women who were not included in the final study population. The Cronbach alphas were 0.85 and 0.75, respectively. The Composite Abuse Scale version 2013 was used to assess the presence of intimate partner violence. The Composite Abuse Scale (CAS) was filled out by the participant after a thorough explanation and understanding of the study instrument. It has earlier been validated in the study area (26).

### Sample size determination

The sample size was calculated using the formula for cross‐sectional study (N=Z^2^ PQ/D^2^) where N is the required sample size, Z is 1.96 at 95% confidence interval (CI), P is estimated patient psychiatric illness prevalence from similar studies; D is the margin of error at 5% (standard deviation of 0.05), and Q is 1 − P. P is 0.306 from previous study (10). A minimum sample size of 326 patients was obtained and, after the addition of 5% attrition rate, it was increased to 342. A total of 684 participants were recruited, with 342 participants assigned to the case group and 342 to the control group.

### Statistical analysis

Data were analyzed using a statistical package for Social Science (IBM SPSS) software (version 26, Chicago II, USA). Continuous variables were presented as mean and standard deviation (Mean ± 2SD), while categorical variables were presented as numbers and proportions. Bivariate and multivariable regressions analyses were performed to interrogate the effect of dependent factors on the independent variable. A difference with a P-value <0.05 was considered statistically significant.

### Ethical clearance

Ethical clearance was obtained from the Health Research and Ethics committee of NAUTH. A signed consent form was obtained from each parturient before recruitment into the study. The study objectives, procedure, and full implications of participation were discussed with the participants before their consent was obtained. The participants were made to understand that declining to participate in the study or withdrawal from the study would have no consequences to obtaining care.

## Results

**Table 1** illustrates the characteristics of the study group. The average age for women in the HIV-positive pregnant women (HPPW) group was 29.9 ± 6.2 years, while for the control group, it was 30.0 ± 6.0 years. A significant number of women (519, 75.7%) had either completed primary or secondary education. Most of the women were employed (450, 65.8%) and were married (650, 95.0%). Additionally, over half (399, 58.3%) of the women studied were in the early stages of pregnancy, at 30 weeks or less. There was no notable difference in the average gestational age between HPPW and the control group (28.6 ± 7.8 vs. 29.2 ± 6.8 weeks; p = 0.255). Women in the HPPW group experienced a greater incidence of IPV compared to their HIV-negative counterparts. The likelihood of any form of IPV occurring is over one in the HPPW group.

**Table 1:** Characteristics of the study population (n = 684).

Parameters	HIV- positive women (n=342)	HIV negative women (n=342)	cOR (95%CI)
Age			
≤30 years	168 (49.1)	176 (51.5)	0.91 (0.67-1.22)
>30 years	174 (50.9)	166 (48.5)	1
Education			
Below tertiary	214 (62.6)	305 (89.2)	0.20 (0.13-0.30)
Tertiary	128 (37.4)	37 (10.8)	1
Marital status			
Married	311 (90.9)	339 (99.1)	0.08 (0.02-0.29)
Un-married	31 (9.1)	3 (0.9)	1
Occupation			
Employed	184 (53.8)	266 (77.8)	0.33 (0.23-0.46)
Unemployed	158 (46.2)	76 (22,2)	1
Residence			
Urban	202 (59.1)	231 (67.5)	0.69 (0.50-0.94)
Rural	140 (40.9)	111 (32.5)	1
Psychological IPV			
Yes	88 (25.7)	3 (0.9)	39.1 (12.2-125.1)
No	254 (74.3)	339 (99.1)	1
Physical IPV			
Yes	80 (23.4)	20 (5.8)	4.91 (2.91-8.23)
No	262 (76.6)	322 (94.2)	1
Sexual IPV			
Yes	110 (32.2)	3 (0.9)	53.5 (16.8-170.7)
No	232 (67.8)	339 (99.1)	1
Parity			
0-3	257 (75.1)	297 (86.8)	0.45 (0.30-0.68)
4-7	85 (24.9)	45 (13.2)	1
Gestational age			
≤30 weeks	199 (58.2)	200 (58.5)	0.98 (0.72-1.33)
>30 weeks	143 (41.8)	142 (41.5)	1

IPV, intimate partner violence.

**Figure 1:**
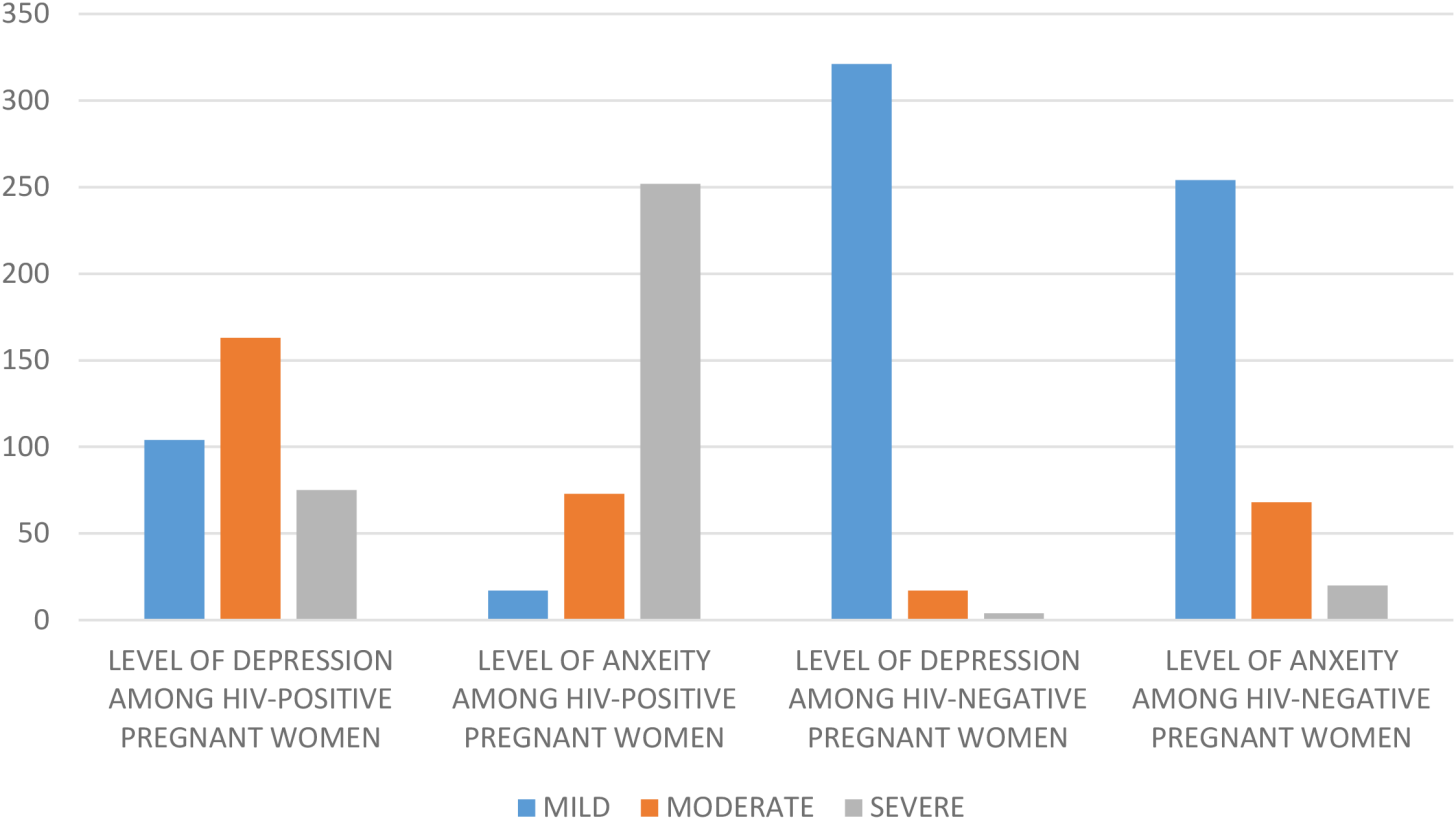
Level of mental health disorder among the study population.

**Figure 1** above represents the level of health disorder among the study population. The average scores for anxiety in HIV-positive and HIV-negative women were 16.8 ± 3.8 compared to 8.7 ± 2.3; P <0.001, while for depression the scores were 11.1 ± 4.3 versus 3.1 ± 3.3; P < 0.001, respectively. A significant presence of major depressive and anxiety disorders was discovered among HIV-positive women, with moderate and severe depression affecting 47.7% and 21.9%, respectively, while moderate and severe anxiety were observed in 21.3% and 73.6% of the women respectively. The majority of women in the control group exhibited mild mental health disorders (not in the table).

**Tables 2**, **3** present a multivariate logistic analysis examining the factors influencing depression and anxiety within the HPPW. All participants in the study exhibited symptoms of anxiety and depression; however, the degree of these symptoms varied among individuals. The rates of moderate and severe depression were found to be 47.7% and 21.9%, respectively, while moderate and severe anxiety affected 21.3% and 73.6% of women respectively. Key predictors of moderate depression within the research group include age, educational attainment, and employment status. Women younger than 30 are 1.8 times more likely to experience moderate depression compared to those aged 30 and older. Those who have only completed primary or secondary education show a higher likelihood of experiencing moderate depression, with the odds being about three times greater in this demographic. Unemployed women face an increased risk of moderate depression. Except for those experiencing psychological IPV, other forms of IPV are correlated with a lower risk of developing moderate depression, as indicated by a negative B coefficient. Women in the earlier stages of pregnancy, specifically those who are 30 weeks or less along, are more likely to endure moderate depression. The research indicated that individuals under 30 years old (AOR 7.43; 95%CI 3.35-16.47, p < 0.001) and those who were at a gestational age of 30 weeks or fewer faced a notably increased risk of suffering from severe depression (AOR 2.86, 95%CI 1.38-5.99, p = 0.005). Women who were married, employed, or had experienced psychological or physical IPV were statistically less likely to suffer from severe depression. Among HPPW, the rates of moderate and severe anxiety were exceptionally high, especially among younger women. Women aged 30 or younger were over 46 times more likely to face moderate or severe anxiety. The group of women who were married, had been exposed to a psychological event, were pregnant, or had given birth to three or fewer children showed significantly lower anxiety levels. While not statistically significant, the presence of sexual IPV demonstrated a very high positive value of B coefficients, indicating a risk of anxiety greater than tenfold. This information is detailed in **Table 3**. The average scores for anxiety in HIV-positive and HIV-negative women were 16.8 ± 3.8 compared to 8.7 ± 2.3; P <0.001, while for depression the scores were 11.1 ± 4.3 versus 3.1 ± 3.3; P < 0.001(not in the tables).

**Table 2:** Multivariate logistic analysis of the determinants of depression among the HIV positive pregnant women (n=342).

Parameters	Level of depression			Moderate depression			Severe depression			
	Mild (n,%)	Moderate (n,%)	Severe (n,%)	B	AOR95%CI	P value	B	AOR 95%CI	P value
Age										
≤30 years	31 (29.8)	81 (49.7)	56 (74.7)	0.59	1.81 (0.99-3.31)	0.050	2.01	7.43 (3.35-16.47)	<0.001
>30 years	73 (70.2)	82 (50.3)	19 (25.3)	R	–		R	–	
Education										
Below tertiary	74 (71.2)	87 (53.4)	53 (70.7)	0.98	2.68 (1.35-5.30)	0.005	0.24	1.28 (0.53-3.09)	0.579
Tertiary	30 (28.8)	76 (46.6)	22 (29.3)	R	–		R	–	
Marital status										
Married	98 (94.2)	150 (92.0)	63 (84.0)	-0.08	0.92 (0.52-1.61)	0.776	-0.86	0.42 (0.21-0.84)	0.015
Un-married	6 (5.8)	13 (8.0)	12 (16.0)	R	–		R	–	
Occupation										
Employed	70 (67.3)	78 (47.9)	36 (48.0)	-0.82	0.44 (0.28-0.78)	0.005	-0.30	0.73 (0.35-1.55)	0.423
Unemployed	34 (32.7)	85 (52.1)	39 (52.0)	R	–		R	–	
Psych. IPV										
Yes	29 (27.9)	43 (26.4)	16 (21.3)	0.01	1.08 (0.32-3.06)	0.988	-0.43	0.64 (0.19-2.16)	0.483
No	75 (72.2)	120 (73.6)	59 (78.7)	R	–		R	–	
Physical IPV										
Yes	25 (24.0)	32 (19.6)	23 (30.7)	-0.38	0.68 (0.36-1.23)	0.205	-0.31	0.73 (0.34-1.54)	0.413
No	79 (76.0)	131 (80.4)	52 (69.2)	R	–		R	–	
Sexual IPV										
Yes	28 (26.9)	49 (30.1)	33 (44.0)	-0.21	0.81 (0.41-1.56)	0.524	0.93	0.39 (0.17-0.90)	0.028
No	76 (73.1)	114 (69.9)	42 (56.0)	R	–		R	–	
Parity										
0-3	61 (58.7)	138 (84.7)	58 (77.3)	-0.08	0.92 (0.46-1.85)	0.823	0.41	1.51 (0.66-3,42)	0.324
4-7	43 (41.3)	25 (15.3)	17 (22.7)	R	–		R	–	
Gestational age										
≤30 weeks	63 (60.6)	101 (62.0)	35 (46.7)	0.47	1.60 (0.86-2.97)	0.137	1.05	2.86 (1.38-5.99)	0.005
>30 weeks	41 (39.4)	62 (38.0)	40 (53.3)	R			R	–	

Mild depression is the reference group. Psych., Psychological.

**Table 3:** Multivariate logistic analysis of the determinants of anxiety among the HIV positive pregnant women (n=342).

Parameters	Level of anxiety			B	Moderate anxiety		B	Severe anxiety	
	Mild	Moderate	Severe		AOR 95%CI	P value		AOR 95%CI	P value
Age									
≤30 years	0 (0.0)	32 (43.8)	136 (54)	46.2	0.03 (0.002-0.62)	0.032	46.4	0.02 (0.001-0.41)	0.009
>30 years	17 (100)	41 (56.2)	116 (46)	R			R		
Education									
Below tertiary	11 (64.7)	43 (58.9)	160 (63.5)	-0.54	0.58 (0.14-2.37)	0.45	-0.71	0.48 (0.13-1.81)	0.28
Tertiary	6 (35.3)	30 (41.1)	92 (36.5)	R			R		
Marital status									
Married	15 (88.2)	71 (97.3)	225 (89.3)	1.36	3.92 (1.05-14.69)	0.04	0.33	1.40 (0.41-4.78)	0.59
Un-married	2 (11.8)	2 (2.7)	27 (10.7)	R			R		
Occupation									
Employed	14 (82.4)	53 (72.6)	117 (46.4)	-0.32	0.72 (0.17-2.99)	0.65	-0.29	0.74 (0.19-2.87)	0.66
Unemployed	3 (17.6)	20 (27.4)	135 (53.6)	R			R		
Psych. IPV									
Yes	2 (11.8)	25 (34.2)	61 (24.2)	32.47	0.25 (0.05-1.20)	<0.001	14.6	0.41 (0.09-1.87)	0.25
No	15 (88.2)	48 (65.8)	191 (75.8)	R			R		
Physical IPV									
Yes	0 (0.0)	25 (34.2)	55 (21.8)	-0.33	0.71 (0.13-3.92)	0.701	-1.49	0.22 (0.04-1.12)	0.99
No	17 (100.0)	48 (65.8)	197 (78.2)	R			R	14	
Sexual IPV									
Yes	2 (11.8)	23 (31.5)	85 (33.7)	15.08	1.99 (0.40-9.81)	0.396	14.68	2.80 (0.61-12.78)	0.18
No	15 (88.2)	50 (68.5)	167 (66.3)	R			R		
Parity									
0-3	12 (70.6)	52 (71.2)	193 (76.6)	17.53	1.03 (0.32-3.29)	<0.001	16.82	1.36 (0.46-4.02)	0.14
4-7	5 (29.4)	21 (28.8)	59 (23.4)	R			R		
Gestational age									
≤30 weeks	7 (41.2)	54 (74.0)	138 (54.8)	0.89	2.45 (0.43-14.00)	0.310	1.24	3.46 (0.65-18.39)	0.06
>30 weeks	10 (58.8)	19 (26.0)	114 (45.2)	R			R		

Mild anxiety is the reference group. Psych., Psychological.

**Tables 4** and **5** represent multivariate logistic analysis of the determinants of depression among the HIV negative women. From the tables, majority of the women had mild depression (93.9%) and is followed by moderate depression (4.9%). Severe depression occurred in 1.2% of the study population. For anxiety disorder, mild anxiety and moderate anxiety occurred in 74.3% and 19.9% of the women respectively. Less than two-third (5.8%) of the study population had severe anxiety. The significant determinates of depression were maternal age, and level of education. Women who were ≤30 years were 1.3 and 2.3 times more likely to develop moderate and severe depression respectively. Even though it was not significant, lower educational attainment, being married and employed were associated with increased odds of developing severe anxiety disorder. Women in their first pregnancy and those with three or less delivery were at lesser risk of developing any form of anxiety disorder because of their negative B coefficient.

**Table 4:** Multivariate logistic analysis of the determinants of depression among the HIV negative women (n=342).

Parameters	Level of Depression			B	Moderate depression		B	Severe depression	
	Mild	Moderate	Severe		AOR 95%CI	P value		AOR 95%CI	P value
Age									
≤30 years	162 (50.5)	14 (82.4)	0 (0.0)	1.29	3.66 (1.10-12.13)	0.03	-2.27	0.10 (0.01-0.56)	0.009
>30 years	159 (49.5)	3 (17.6)	4 (100.0)	R			R		
Education									
Below tertiary	284 (88.5)	17 (100.0)	4 (100.0)	-1.95	0.14 (0.04-0.48)	0.002	2.63	13.86 (0.57-332.95)	0.105
Tertiary	37 (11.5)	0 (0.0)	0 (0.0)	R			R		
Marital status									
Married	321 (100.0)	14 (82.4)	4 (100.0)	0.77	2.16 (0.77-6.06)	0.141	-2.07	0.12 (0.02-0.65)	0.013
Un-married	0 (0.0)	3 (17.6)	0 (0.0)	R			R		
Occupation									
Employed	248 (77.3)	14 (82.4)	4 (100.0)	0.95	2.59 (0.24-27.86)	0.43	1.53	4.62 (0.13-163.38)	0.400
Unemployed	73 (22.7)	3 (17.6)	0 (0.0)	R			R		
Psych. IPV									
Yes	3 (0.9)	0 (0.0)	0 (0.0)	-20.32	3.88 (0.18-79.7)	0.37	-2.40	1.0 (0.04-22.7)	1.00
No	318 (99.1)	17 (100.0)	4 (100.0)	R			R		
Physical IPV									
Yes	20 (6.2)	0 (0.0)	0 (0.0)	1.10	3.02 (0.62-14.66)	0.16	1.27	3.58 (0.49-25.98)	0.206
No	301 (93.8)	17 (100.0)	4 (100.0)	R			R		
Sexual IPV									
Yes	0 (0.0)	3 (17.6)	0 (0.0)	-1.57	0.06 (0.003-1.31)	0.07	1.27	0.13 (0.002-7.88)	0.337
No	321 (100.0)	14 (82.4)	4 (100.0)	R	4		R		
Parity									
0-3	281 (87.5)	12 (70.6)	4 (100.0)	-0.38	0.67 (0.03-13.43)	0.79	-1.2	0.30 (0.004-22.96)	0.559
4-7	40 (12.5)	5 (29.4)	0 (0.0)	R			R		
GA									
≤30 weeks	186 (57.9)	14 (82.4)	0 (0)	0.77	2.16 (0.77-6.06)		-2.70	0.12 (0.02-0.65)	0.01
>30 weeks	135 (42.1)	3 (17.6)	4 (100)	R			R		

Mild anxiety is the reference group. Psych., Psychological.

**Table 5:** Multivariate logistic analysis of the determinants of anxiety among HIV negative women (n=342).

Parameters	Level of Anxiety			B	Moderate anxiety		B	Severe anxiety	
	Mild	Moderate	Severe		AOR 95%CI	P value		AOR 95%CI	P value
Age									
≤30 years	144 (56.7)	40 (58.8)	16 (80.0)	-0.53	0.58 (0.32-1.03)	0.06	1.03	2.80 (0.95-9.30)	0.06
>30 years	110 (43.3)	28 (41.2)	4 (20.0)	R			R		
Education									
Below tertiary	221 (87.0)	65 (95.6)	19 (95.0)	0.29	1.34 (0.60-2.96)	0.46	0.64	1.91 (0.25-14.16)	0.52
Tertiary	33 (13.0)	3 (4.4)	1 (5.0)	R			R		
Marital status									
Married	254 (100)	68 (100.0)	17 (85.0)	0.29	1.33 (0.76-2.32)	0.30	0.64	1.91 (0.71-5.13)	0.19
Un-married	0 (0.0)	0 (0.0)	3 (15.0)	R			R		
Occupation									
Employed	194 (76.4)	61 (89.7)	11 (55.0)	1.13	3.11 (1.04-9.25)	0.04	0.84	2.33 (0.40-13.37)	0.34
Unemployed	60 (23.6)	7 (10.3)	9 (45.0)	R			R		
Psych. IPV									
Yes	3 (1.2)	0 (0.0)	0 (0.0)	-0.48	0.61 (0.61-0.73)	–	-15.85	1.30 (0.55 – 0.68)	0.98
No	251 (100.0)	68 (100.0)	20 (100.0)	R			R		
Physical IPV									
Yes	20 (7.9)	0 (0.0)	0 (0.0)	0.75	2.11 (0.96-4.64)	0.06	-0.44	0.64 (0.24-1.66)	0.36
No	234 (92.1)	68 (100.0)	20 (100.0)	R			R		
Sexual IPV									
Yes	0 (0.0)	0 (0.0)	3 (15.0)	-0.16	0.026 (0.005-13.68)	0.521	-0.56	0.009 (0.005-3.68)	0.002
No	254 (100.0)	68 (100.0)	17 (85.0)	R			R		
Parity									
0-3	220 (86.6)	57 (83.8)	20 (100.0)	-1.45	0.23 (0.03-1.60)	0.14	-1.59	0.20 (0.007-5.98)	0.35
4-7	34 (13.4)	11 (16.2)	0 (0.0)	R			R		
GA									
≤30 weeks	144 (56.7)	40 (58.8)	16 (80.0)	0.29	1.33 (0.76-2.32)	0.30	0.64	1.91 (0.71-5.13)	0.19
>30 weeks	110 (43.3)	28 (41.2)	4 (20)	R			R		

Mild anxiety is the reference group. Psych., Psychological.

## Discussion

Mental health challenges during pregnancy significantly affect both the health of the mother and the newborn. The purpose of the research is to assess the prevalence of mental disorders and the factors that influence them among pregnant patients, both HIV-positive and HIV-negative, at the Nnamdi Azikiwe University Teaching Hospital in Nnewi. All individuals showed signs of anxiety and despair, with HIV-positive pregnant women exhibiting a much greater incidence. Among HIV-positive pregnant women, the rates for mild, moderate, and severe depression and anxiety were 30.4%, 47.7%, and 21.9%, and 4.9%, 21.3%, and 73.6%, respectively. On the other hand, in the control group, the percentages of mild, moderate, and severe depression and anxiety were 93.9%, 4.9%, and 1.2%, and 74.3%, 19.9%, and 5.8%, respectively. In HPPW, maternal age, educational attainment, employment status, marital status, and gestational age emerged as important predictors of depression, while marital status, number of children, and gestational age were identified as significant predictors of anxiety. Among women who are HIV-negative, the levels of depression were primarily influenced by age, marital status, and level of education, while maternal age and job status served as predictors of anxiety.

The occurrence of mental disorders during pregnancy varies across the globe. A comprehensive review conducted by Boakye et al. (7) found that the rates of depression and anxiety in women with HIV in sub-Saharan Africa ranged from 5.9% to 61% for depression and from 28.9% to 61% for anxiety disorders. This prevalence significantly highlights the issue within the obstetric population. The findings noted above align with our results, as all participants in our study exhibited some form of mental disorder. In a related study conducted in Nigeria, Akinsolu et al. found a significant occurrence of depression among women living with HIV, with 69% of these women reporting depressive symptoms (6). A study conducted in Ethiopia among HIV-positive pregnant women found that the prevalence of depression and anxiety was 32.5% and 28.9%, respectively (9). Additionally, 22.7% of patients had concomitant conditions (9). The prevalence of mild, moderate, and severe depression was 18.2%, 12%, and 1.7%, respectively in their study (9). In contrast, our study indicated a notably higher percentage of participants reporting different levels of depression. The prevalence of moderate and severe depression observed in our study population is quite significant, highlighting the manifestation of major depression among HIV-positive pregnant women in the area. This depression may lead to virological failure due to non-adherence to Highly active antiretroviral therapy (HAART) (27), thus heightening the risk of vertical transmission (28). In our research, we found that the rate of anxiety is significantly elevated among women living with HIV. The majority of these women experience severe anxiety. Particularly, severe anxiety is linked to reduced gestation periods and preterm delivery (29). This can have detrimental impacts on prenatal neurodevelopment and negative results for the child (29). A prominent form of anxiety is referred to as ‘pregnancy anxiety.’ ‘Pregnancy anxiety’ represents a notable type of anxiety that occurs as a unique syndrome characterized by concerns about one’s health and survival during pregnancy, the health and welfare of the unborn child, experiences related to hospitals and healthcare, parenting, or fulfilling the role of a mother (28). In contrast to the significant rates of severe depression and anxiety observed in pregnant women living with HIV, women in the control group fared better in terms of disease severity. In our research, the majority of HIV-negative women experienced mild levels of depression and anxiety, which aligns with expectations since HIV infection is linked to a higher incidence of affective disorders. The underlying causes are not fully understood, but there is a connection to central nervous system (CNS) disorders induced by HIV. Some of the pathological changes in the CNS that are related to mental health issues include disturbances in microglial signaling pathways associated with apoptosis, HIV-related dementia, neurocognitive disorders linked to HIV-1 due to neuronal damage, death of oligodendrocytes, and neurodegenerative conditions impacting neurons.

In our study, pregnant women with HIV who are 30 years old or younger had a greater probability of developing severe depressive disorders, with an odds ratio that exceeded one. This result is concerning, as elevated depression levels in this at-risk population could negatively affect their quality of life. This situation may also increase the risk of suicidal thoughts (20). Our observation of a notable link between maternal factors and depression was not corroborated by the findings of Yousuf et al. in Ethiopia (9). Variations in the study population may explain the results observed. Additionally, **Table 2** clearly indicates that obtaining a tertiary education lowers the likelihood of experiencing major depression. One possible explanation for this is that a higher level of education enhances women’s capacity to manage the stress associated with HIV. The development of self-control or escape-avoidance coping strategies, which can be linked to higher education levels, may clarify our findings. In our study, married women and those who were employed showed resilience against major depressive illness. This finding aligns with a previous study by Abebe et al. (10), which indicated that the likelihood of experiencing antenatal depression was three times greater among unmarried HIV-positive pregnant women compared to their married counterparts. This effect can be linked to the support provided by family and the empowerment that comes from enhanced economic conditions for women. Research indicates that social support can enhance psychological resilience in women living with HIV, serving as a mediator in decreasing depression. A concerning observation from our study is the early occurrence of major depressive disorder during pregnancy among our cohort of HIV-positive pregnant women. This highlights the need for prenatal care providers in the region to establish a protocol for the early screening of depression in HIV-positive pregnant individuals. Timely identification will facilitate prompt management. The early appearance of psychological distress during pregnancy may be associated with prior depressive illnesses that occurred before pregnancy, experiences with intimate partner violence (IPV), or feelings of stigmatization. IPV could be a contributing factor, as 24.1% of the women reported being victims of it. Among HIV-negative women, most experienced mild depression, with significant factors influencing these outcomes being maternal age, marital status, and educational attainment. Like HIV-positive women, moderate depression is more prevalent among younger women, but there is an inverse relationship with maternal age regarding severe depression. Women under 30 years of age were less likely to experience severe depression (B = -2.27; AOR = 0.10 95%CI 0.01-0.56; P = 0.009). This contrasts with the findings for HIV-positive women previously discussed. Additionally, our study indicates that lower levels of education are a risk factor for severe depression. In our research, the prevalence of anxiety disorders was notably high among HIV-positive individuals. This aligns with previous studies (9, 30–32). The significant increase in severe anxiety disorders is particularly evident in women aged 30 and under, those with three or fewer children, and those who have experienced both physical and psychological violence from intimate partners. The likelihood of experiencing anxiety is reduced in women with lower educational levels, while employment serves as a protective factor, as one would anticipate. Nevertheless, the coping mechanisms of these women, similar to those of HIV-positive women, may be a recurring aspect of our findings. In comparison to the cases, the control group exhibited a lower burden of the condition, with most individuals experiencing only mild anxiety. Besides occupational status having a significant impact on the prevalence of moderate anxiety, the other variables examined in our study were not significantly linked to the occurrence of either moderate or severe anxiety. Additionally, the unit (B) change in the burden of moderate or severe anxiety is lower for HIV-negative women compared to those with HIV, in relation to predictor variables like the participant’s age, parity, and experiences of psychological and sexual IPV. Our results align with previous research (33). Regardless of severity, anxiety during pregnancy is connected to complications for both the mother and the newborn, as previously discussed. It appears that mild anxiety is associated with fewer complications. In individuals infected with HIV, anxiety is linked to a delay in starting antiretroviral therapy (27), which can result in virological failure and subsequently increase the risk of vertical transmission (28). In a similar study conducted during the COVID-19 pandemic, Ade-Ojo et al. noted that the group living with HIV exhibited higher rates of major depressive disorder and severe anxiety disorder compared to the HIV-negative group (34).

## Conclusion

In summary, the occurrence of mental health disorders is significantly high among the participants in the study. Women living with HIV experienced a greater severity of illness compared to those who were HIV negative. The incidence of depression (HPPW) was notably higher in women aged 30 or younger, those with lower educational levels, and those who were at or below 30 weeks of gestation, whereas anxiety disorders were more prevalent among married women and those who had experienced psychological and sexual intimate partner violence (IPV). In contrast, most HIV-negative women reported mild mental health issues. The study indicated that HIV-negative women under 30 and who are married were less prone to severe depression. IPV emerged as a significant predictor for the onset of depression in HIV-negative women.

### Study limitations

As our research was carried out within a hospital setting, the findings may not be applicable to individuals attending prenatal care in the region analyzed. Nevertheless, the substantial number of participants involved in our study, which served as a measure of quality control, allowed for a significant assessment of the burden of maternal depression in the area of study. There is a possibility that social desirability bias may have influenced the results of our study. To mitigate the risk of stigmatization, we ensured that the questionnaire was kept anonymous. Counseling services were also offered, and participants were clearly informed that their responses would not affect the quality of care they received and that they had the right to withdraw from the study at any time without any negative consequences. We opted for screening tools for depression and anxiety instead of conducting clinical interviews. Non-probability sampling method was used for participant selection which introduce bias but this bias was mitigated the large sample size and the matching procedure employed in the selection of the control. It is also important to note that the small count reported in some of the cells might have an unstable effect on the odds ratio generated. This effect can be eliminated by conducting a multicenter study with a larger sample size.

### Recommendation

The burden of mental health disorder is very high among the study population, especially among the HIV-positive pregnant women. This finding is unacceptable, and it demands the development of a protocol for universal screening of the presence of depression and anxiety disorders among antenatal attendees in the hospital and by extension, the study area. This is especially important among pregnant women living with HIV. It is important that pregnant women should be encouraged to speak out and seek help from their health care providers. The services of a clinical psychologist is also needed during antenatal care services to help provide health talks, counselling and treatment for an affected woman.

## Data Availability

The original contributions presented in the study are included in the article/supplementary material. Further inquiries can be directed to the corresponding author.

## References

[1] World Health Organization . Anxiety disorders. In: Anxiety Disorders (2023). Available online at: https://www.who.int/news-room/fact-sheets/detail/anxiety-disorders (Accessed July 7, 2024).

[2] World Health Organization . Depressive disorder (2023). Available online at: https://www.who.int/news-room/fact-sheets/detail/depression.

[3] World Health Organization . Mental Health, Brain Health, Substance Use (Maternal Mental Health). Available online at: https://www.who.int/teams/mental-health-and-substance-use/promotion-prevention/maternal-mental-health.

[4] Zhu Q-Y Huang D-S Lv J-D Guan P Bai X-H . Prevalence of perinatal depression among HIV-positive women: a systematic review and meta-analysis. BMC Psychiatry. (2019) 19:330. doi: 10.1186/s12888-019-2321-2, PMID: 31666033 PMC6822469

[5] Remien RH Stirratt MJ Nguyen N Robbins RN Pala AN Mellins CA . Mental health and HIV/AIDS. AIDS. (2019) 33:1411–20. doi: 10.1097/QAD.0000000000002227, PMID: 30950883 PMC6635049

[6] Akinsolu FT Abodunrin OR Lawale AA Bankole SA Adegbite ZO Adewole IE . Depression and perceived stress among perinatal women living with HIV in Nigeria. Front Public Health. (2023) 11:1259830. doi: 10.3389/fpubh.2023.1259830, PMID: 38054071 PMC10694505

[7] Boakye DS Setordzi M Dzansi G Adjorlolo S . Mental health burden among females living with HIV and AIDS in sub-Saharan Africa: A systematic review. PloS Global Public Health. (2024) 4(2):e0002767. doi: 10.1371/journal.pgph.0002767, PMID: 38300927 PMC10833589

[8] Madundo K Knettel BA Knippler E Mbwambo J . Prevalence, severity, and associated factors of depression in newly diagnosed people living with HIV in Kilimanjaro, Tanzania: a cross-sectional study. BMC Psychiatry. (2023) 23:83. doi: 10.1186/s12888-022-04496-9, PMID: 36726113 PMC9890688

[9] Yousuf A Musa R Isa ML Arifin SRM . Anxiety and depression among women living with HIV: prevalence and correlations. Clin Pract Epidemiol Ment Health. (2020) 16:59–66. doi: 10.2174/1745017902016010059, PMID: 32742296 PMC7372730

[10] Abebe GF Alie MS Adugna A Asemelash D Tesfaye T Girma D . Antenatal depression and its predictors among HIV positive women in Sub-Saharan Africa; a systematic review and meta-analysis. Front Psychiatry. (2024) 15:1385323. doi: 10.3389/fpsyt.2024.1385323, PMID: 38919635 PMC11196764

[11] Bante A Mersha A Zerdo Z Wassihun B Yeheyis T . Comorbid anxiety and depression: Prevalence and associated factors among pregnant women in Arba Minch zuria district, Gamo zone, southern Ethiopia. PLoS One. (2021) 16:e0248331. doi: 10.1371/journal.pone.0248331, PMID: 33690693 PMC7946223

[12] Takelle GM Nakie G Rtbey G Melkam M . Depressive symptoms and associated factors among pregnant women attending antenatal care at Comprehensive Specialized Hospitals in Northwest Ethiopia 2022: an institution-based cross-sectional study. Front Psychiatry. (2023) 14:1148638. doi: 10.3389/fpsyt.2023.1148638, PMID: 37415690 PMC10322208

[13] Thompson O Ajayi I . Prevalence of antenatal depression and associated risk factors among pregnant women attending antenatal clinics in Abeokuta north local government area, Nigeria. Depression Res Treat. (2016) 2016:1–15. doi: 10.1155/2016/4518979, PMID: 27635258 PMC5007324

[14] Wang Y Wang X Liu F Jiang X Xiao Y Dong X . Negative life events and antenatal depression among pregnant women in Rural China: the role of negative automatic thoughts. PLoS One. (2016) 11:e0167597. doi: 10.1371/journal.pone.0167597, PMID: 27977715 PMC5157981

[15] Amu H Sanjay Sane S Xue Y . Global, regional, and national HIV/AIDS disease burden levels and trends in 1990–2019: A systematic analysis for the global burden of disease 2019 study (1980). Available online at: http://ghdx.healthdata.org/ (Accessed July 7, 2024). 10.3389/fpubh.2023.1068664 PMC9975742 36875364

[16] Glover V . Maternal depression, anxiety and stress during pregnancy and child outcome; What needs to be done. Best Pract Research: Clin Obstetrics Gynaecology. (2014) 28:25–35. doi: 10.1016/j.bpobgyn.2013.08.017, PMID: 24090740

[17] Kitai T Komoto Y Kakubari R Konishi H Tanaka E Nakajima S . A comparison of maternal and neonatal outcomes of pregnancy with mental disorders: results of an analysis using propensity score-based weighting. Arch Gynecology Obstetrics. (2014) 290:883–9. doi: 10.1007/s00404-014-3304-7, PMID: 24927782

[18] Suman V Luther EE . Preterm labor. In: StatPearls, vol. 2024. StatPearls Publishing, Treasure Island (FL (2023).

[19] VanderKruik R Barreix M Chou D Allen T Say L Cohen LS . The global prevalence of postpartum psychosis: A systematic review. BMC Psychiatry. (2017) 17(1):272. doi: 10.1186/s12888-017-1427-7, PMID: 28754094 PMC5534064

[20] Bete T Ali T Misgana T Negash A Abraham T Teshome D . Suicidal ideation and associated factors among pregnant women attending antenatal care at public hospitals of Harari regional state, eastern Ethiopia: A cross-sectional study. PLoS One. (2024) 19(3):e0300417. doi: 10.1371/journal.pone.0300417, PMID: 38547179 PMC10977762

[21] Awofala AA Ogundele OE . HIV epidemiology in Nigeria. Saudi J Biol Sci. (2018) 25:697–703. doi: 10.1016/j.sjbs.2016.03.006, PMID: 29740232 PMC5937013

[22] Bassey AE Miteu GD . A review of current trends in HIV epidemiology, surveillance, and control in Nigeria. Ann Med Surg. (2023) 85:1790–5. doi: 10.1097/MS9.0000000000000604, PMID: 37229028 PMC10205236

[23] Tian X Chen J Wang X Xie Y Zhang X Han D . Global, regional, and national HIV/AIDS disease burden levels and trends in 1990–2019: A systematic analysis for the global burden of disease 2019 study. Front Public Health. (2023) 11:1068664. doi: 10.3389/fpubh.2023.1068664, PMID: 36875364 PMC9975742

[24] Kroenke K Spitzer RL Williams JBW . The PHQ-9. J Gen Internal Med. (2001) 16:606–13. doi: 10.1046/j.1525-1497.2001.016009606.x, PMID: 11556941 PMC1495268

[25] Sapra A Bhandari P Sharma S Chanpura T Lopp L . Using generalized anxiety disorder-2 (GAD-2) and GAD-7 in a primary care setting. Cureus. (2020) 12(5):e8224. doi: 10.7759/cureus.8224, PMID: 32582485 PMC7306644

[26] Anikwe CC Umeononihu OS Anikwe IH Ikeoha CC Eleje GU Ewah RL . Burden of intimate partner violence among nurses and nursing students in a tertiary hospital in Abakaliki, Ebonyi state, Nigeria. SAGE Open Nurs. (2021) 7:23779608211052356. doi: 10.1177/23779608211052356, PMID: 34869862 PMC8640327

[27] Rane MS Hong T Govere S Thulare H Moosa M-Y Celum C . Depression and anxiety as risk factors for delayed care-seeking behavior in human immunodeficiency virus–infected individuals in South Africa. Clin Infect Dis. (2018) 67:1411–8. doi: 10.1093/cid/ciy309, PMID: 29659757 PMC6186861

[28] Concepcion T Velloza J Kemp CG Bhat A Bennett IM Rao D . Perinatal depressive symptoms and viral non-suppression among a prospective cohort of pregnant women living with HIV in Nigeria, Kenya, Uganda, and Tanzania. AIDS Behav. (2023) 27:783–95. doi: 10.1007/s10461-022-03810-6, PMID: 36210392 PMC9944362

[29] Dunkel Schetter C Tanner L . Anxiety, depression and stress in pregnancy: Implications for mothers, children, research, and practice. Curr Opin Psychiatry. (2012) 25:141–8. doi: 10.1097/YCO.0b013e3283503680, PMID: 22262028 PMC4447112

[30] Jalal SM Alsebeiy SH Alshealah NMJ . Stress, anxiety, and depression during pregnancy: A survey among antenatal women attending primary health centers. Healthcare (Switzerland). (2024) 12(22):2227. doi: 10.3390/healthcare12222227, PMID: 39595425 PMC11593483

[31] Meza-Rodríguez M del P Farfan-Labonne B Avila-García M Figueroa-Damian R Plazola-Camacho N . Psychological distress, anxiety, depression, stress level, and coping style in HIV-pregnant women in Mexico. BMC Psychol. (2023) 11:366. doi: 10.1186/s40359-023-01416-8, PMID: 37915068 PMC10621089

[32] Qin S Tan Y Lu B Cheng Y Nong Y . Survey and analysis for impact factors of psychological distress in HIV-infected pregnant women who continue pregnancy. J Maternal-Fetal Neonatal Med. (2024) 32:3160–7. doi: 10.1080/14767058.2018.1459550, PMID: 29764247

[33] Nosik M Lavrov V Svitich O . HIV infection and related mental disorders. Brain Sci. (2021) 11:248. doi: 10.3390/brainsci11020248, PMID: 33671125 PMC7922767

[34] Ade-Ojo IP Dada MU Adeyanju TB . Comparison of anxiety and depression among HIV-positive and HIV-negative pregnant women during COVID-19 pandemic in Ekiti State, southwest Nigeria. Int J Gen Med. (2022) 15:4123–30. doi: 10.2147/IJGM.S362225, PMID: 35465305 PMC9020505

